# Pigmentary Demarcation Lines During Pregnancy With Erythema

**DOI:** 10.7759/cureus.46023

**Published:** 2023-09-26

**Authors:** Oscar V Navea, Maria B Navea, Raul De la Fuente, Marta Valenzuela

**Affiliations:** 1 General Practice, Universidad de los Andes, Santiago, CHL; 2 General Practice, Universidad de Chile, Santiago, CHL; 3 Dermatology, Hospital Clínico de la Universidad de Chile, Santiago, CHL; 4 Dermatology, Clinica Prima Piel, Santiago, CHL

**Keywords:** type-b pigmentary demarcation lines, pigmentary disorders, pregnancy associated erythema, voigt-futcher lines, pigmentary demarcation lines

## Abstract

Pigmentary demarcation lines (PDL), or Voigt-Futcher lines, are lines that mark an abrupt transition between hyperpigmented skin and normal skin. PDLs are more common in Japanese and dark-skinned individuals. Eight types have been described (A-H); Type B is located on the posteromedial aspect of the lower extremities; it is more common in women and is the one most frequently associated with pregnancy. The demarcation lines of pregnancy are of unknown etiology; they appear mainly in the last trimester and disappear spontaneously months after delivery.

We report a case of pregnancy-associated PDL with erythema without melanocytic pigmentation in a 23-week-gestational Latin primiparous woman.

## Introduction

Pigmentary demarcation lines (PDL) or Voigt-Futcher lines are abrupt physiological transitions between hyperpigmented and lighter skin. The pathogenesis is unknown, and eight types have been described according to their anatomical location (A-H) [1]. The PDLs correspond to certain Voigt lines, which delimit the distribution of peripheral cutaneous nerves. They are more frequent in women and in Black and Japanese populations [1,2].

In pregnant women, the most commonly associated is Type B. This occurs along the posterior aspect of the lower extremity and is generally bilateral and symmetrical [1]. There are also reports of the concomitance of PDL Type A and B [3,4]. Type A PDL occurs on the anterolateral aspect of the upper arms and extends over the pectoral area [5].

Most PDLs appear in the last trimester of pregnancy and resolve spontaneously after delivery in a period of 6 to 12 months [2,6].

In pregnant women, cases of Type B PDL with erythema have been reported [2,7] and Type B with erythema and involvement of the anterior aspects of the thighs and knees [8].

The histology of PDL Type B is variable, showing a slight infiltration of lymphocytes around the vessels in the upper dermis with mild hyperpigmentation of the basal layer. In another study, basal and suprabasal pigmentation was described with mild pigment incontinence in the papillary dermis, and there may be only increased melanization [1].

## Case presentation

**Figure 1:**
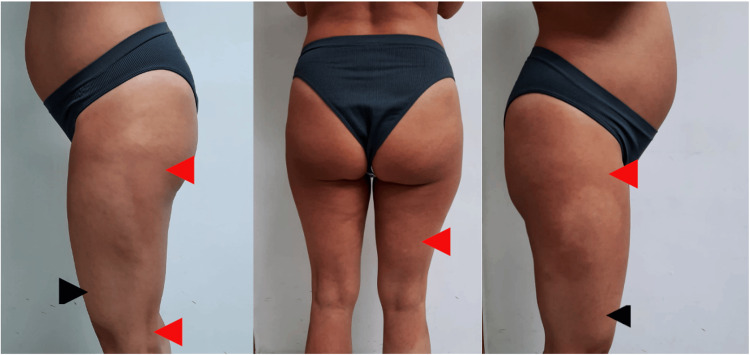
Clinical pictures Clinical pictures show an erythematous, well-defined area (red arrows) that compromises from the root of the thighs and lower pubic area to the upper limit of the knees; from the lower portion of the buttocks to the proximal third of the calves; and on the lateral aspects, a strip on the upper portion of the thighs that connects the anterior maculae with the posterior ones. Black arrows show normal skin.

**Figure 2:**
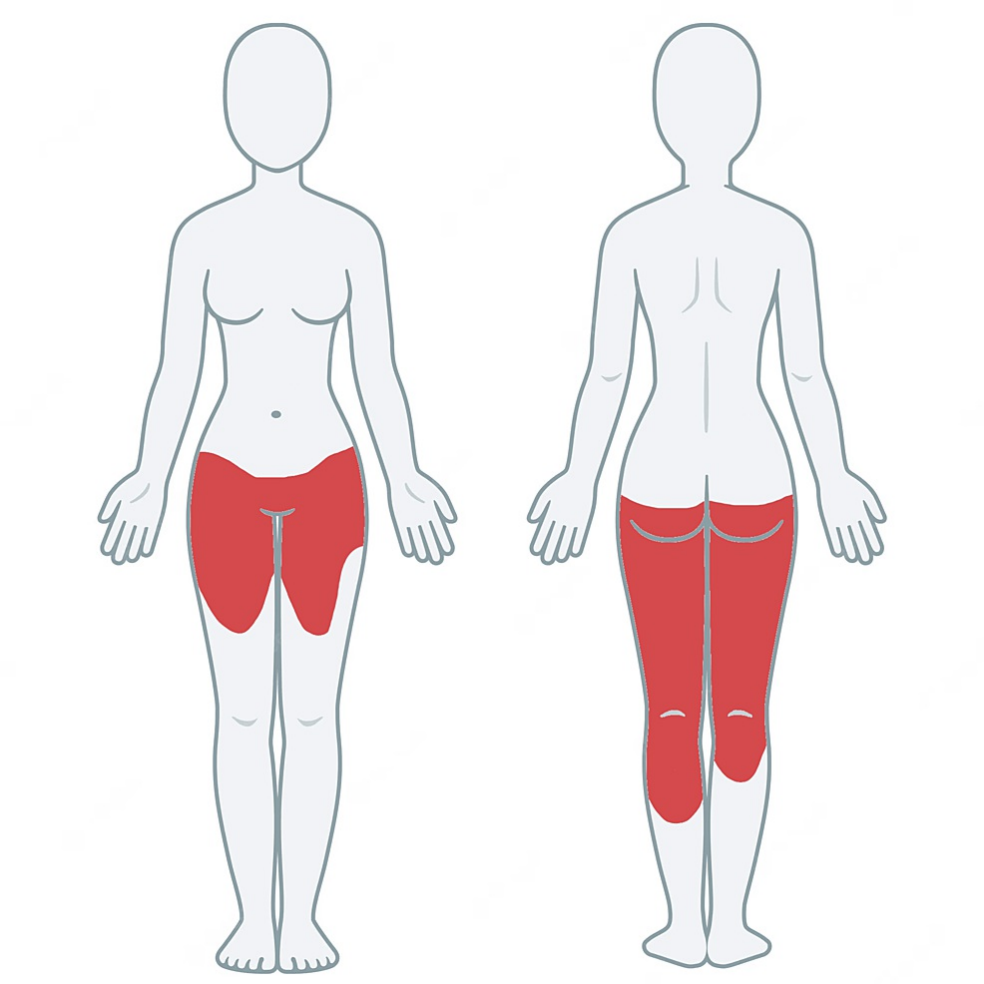
Patient diagram The diagram shows the affected area in red.

A healthy, 33-year-old Latin woman, with Fitzpatrick phototype III, 23 weeks pregnant with a normal pregnancy, presents an erythematous, well-defined, symmetrical, and extensive area that is asymptomatic, with a two-week evolution. The affected area compromises from the root of the thighs and lower pubic area to the upper limit of the knees, from the lower portion of the buttocks to the proximal third of the calves, and on the lateral aspects, a strip on the upper portion of the thighs that connects the anterior maculae with the posterior ones (Figures 1-2). When compressing the affected area, positive diascopy was observed without residual pigmentation. The patient was undergoing treatment with vitamins and calcium, and laboratory studies were normal. Color Doppler ultrasonography of the lower extremities with perforator vein mapping resulted in no pathological findings.

**Figure 3:**
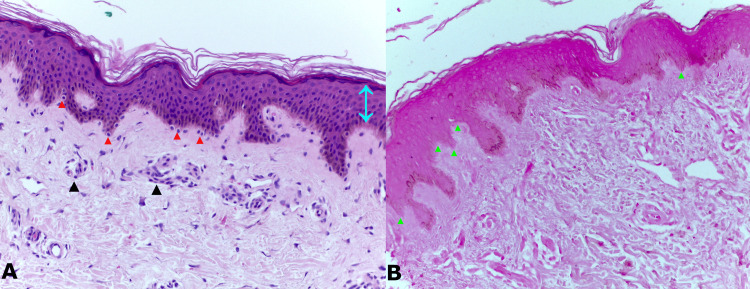
Histopathological images (A) 200X H&E stain shows orthokeratosis, preserved epidermis (blue arrow), presence of melanocytes in the basal layer (red arrows), in quantity and distribution within the normal range, absence of melanophages in the superficial dermis, and mild superficial perivascular lymphocytic infiltrate (black arrows). (B) 200X Fontana Masson stain shows areas of basal epidermal hypomelanosis (green arrows).

A biopsy of a thigh lesion was performed, which described orthokeratosis, preserved epidermis, presence of melanocytes in the basal layer, in quantity and distribution within the normal range, absence of melanophages in the superficial dermis, and mild superficial perivascular lymphocytic infiltrate. With Fontana Masson staining, areas of basal epidermal hypomelanosis were observed (Figure 3).

After delivery, lesions resolved spontaneously without residual pigmentation over a period of four weeks.

## Discussion

From the first descriptions of PDL, reference is made both in clinical and histological findings to melanin-type pigmentation [9,10]. In 1984, Type B PDL associated with pregnancy was first reported. The authors described two cases, one of them a White woman in whom the pigmented skin biopsy showed mild hyperpigmentation of the basal layer [11]. In 1985, a case of PDL was reported in a pregnant Japanese woman. This is the first report that highlights the coexistence of melanin pigmentation and erythema, in addition to a distribution different from the classic PDL Type A and B described, because it compromised the inguinal and pubic areas, as well as the anterior brachial portion with transpectoral extension to the breasts. They observed pigmentation under the positive diascopy, and postpartum, the erythema disappeared before the pigmentation [12]. In 2012, a case of Type B PDL associated with pregnancy and erythema was reported, in which it also compromised the anterior aspects of the thighs up to the knees. The histological study showed pigmentation in the basal layer and dilation of small vessels with perivascular lymphohistiocytic infiltrate in the upper dermis [8].

The case that we report also presents differences from the majority of the previously reported cases. It occurs in a Latin woman (skin phototype III), and the PDLs appeared at 21 weeks of gestation, clinically only in the presence of erythema and histologically without melanin pigmentation. In addition, the anatomical distribution in the lower extremities is more extensive than previously reported.

## Conclusions

PDLs associated with pregnancy are rare, self-limited, and still of unknown etiology. Most reported cases correspond to Type B PDLs with melanin pigmentation. However, new cases with variations in distribution, type of pigment, and histopathological findings make us think that they could correspond to another entity. We consider it important that obstetricians and dermatologists are aware of this condition for future research.
